# Patient-reported outcomes from a randomized phase II study of the deferasirox film-coated tablet in patients with transfusion-dependent anemias

**DOI:** 10.1186/s12955-018-1041-5

**Published:** 2018-11-19

**Authors:** Ali T. Taher, Raffaella Origa, Silverio Perrotta, Alexandra Kouraklis, Giovan Battista Ruffo, Antonis Kattamis, Ai-Sim Goh, Vicky Huang, Aiesha Zia, Raquel Merino Herranz, John B. Porter

**Affiliations:** 1 Department of Internal Medicine, Director – Fellowship and Residents Research Program, Faculty of Medicine American University of Beirut Medical Center, Beirut, Lebanon; 2 Ospedale Pediatrico Microcitemico ‘A.Cao’, A.O. ‘G. Brotzu’, Cagliari, Italy; 3 Università della Campania “L. Vanvitelli,”, Caserta, Italy; 4 Hematology Division, Department of Internal Medicine, University of Patras Medical School, Patras, Greece; 5 U.O.C. Ematolog. Con Talassemia, A.O. Civico-Di Cristina-Benfratelli, Palermo, Italy; 6 First Department of Pediatrics, University of Athens, Athens, Greece; 7 Department of Medicine, Hospital Pulau Pinang, Georgetown, Penang Malaysia; 8 Novartis Pharmaceuticals Corporation, East Hanover, NJ USA; 9 Novartis Pharma AG, Basel, Switzerland; 10 University College London, London, UK; 11 Emory School of Medicine, Atlanta, USA

**Keywords:** Iron chelation, Iron overload, Deferasirox, Patient-reported outcomes

## Abstract

**Background:**

Adherence to long-term chelation therapy in transfusion-dependent patients is critical to prevent iron overload-related complications. Once-daily deferasirox dispersible tablets (DT) have proven long-term efficacy and safety in patients ≥2 years old with chronic transfusional iron overload. However, barriers to optimal adherence remain, including palatability, preparation time, and requirements for fasting state. A new film-coated tablet (FCT) formulation was developed, swallowed once daily (whole/crushed) with/without a light meal.

**Methods:**

The open-label, Phase II ECLIPSE study evaluated patient-reported outcomes (PROs) in transfusion-dependent thalassemia or lower-risk myelodysplastic syndromes patients randomized 1:1 to receive deferasirox DT or FCT over 24 weeks as a secondary outcome of the study. Three PRO questionnaires were developed to evaluate both deferasirox formulations: 1) Modified Satisfaction with Iron Chelation Therapy Questionnaire; 2) Palatability Questionnaire; 3) Gastrointestinal (GI) Symptom Diary.

**Results:**

One hundred seventy three patients were enrolled; 87 received the FCT and 86 the DT formulation. FCT recipients consistently reported better adherence (easier to take medication, less bothered by time to prepare medication and waiting time before eating), greater satisfaction/preference (general satisfaction and with administration of medicine), and fewer concerns (less worry about not swallowing enough medication, fewer limitations in daily activities, less concern about side effects). FCT recipients reported no taste or aftertaste and could swallow all their medicine with an acceptable amount of liquid. GI summary scores were low for both formulations.

**Conclusions:**

These findings suggest a preference in favor of the deferasirox FCT formulation regardless of underlying disease or age group. Better patient satisfaction and adherence to chelation therapy may reduce iron overload-related complications.

**Trial registration:**

ClinicalTrials.gov identifier: NCT02125877; registered April 26, 2014.

**Electronic supplementary material:**

The online version of this article (10.1186/s12955-018-1041-5) contains supplementary material, which is available to authorized users.

## Background

In patients with hematological disorders requiring red blood cell transfusions to treat anemia, such as those with transfusion-dependent thalassemia (TDT) and myelodysplastic syndromes (MDS), long-term iron chelation therapy is necessary to remove excess iron and prevent organ failure [1]. Because iron overload is a chronic condition and the benefits of chelation therapy are not immediately perceivable, compliance with chelation therapy has a positive influence on the occurrence of iron overload-related complications and survival [2–4]. As such, a convenient and well-tolerated chelation regimen is required for optimal patient outcomes.

The once-daily oral deferasirox dispersible tablet (DT) formulation (Exjade®) has been used in clinical practice for over a decade, with a well-established efficacy and safety profile in patients ≥2 years of age with chronic transfusional iron overload [5–13]. Oral deferasirox DT offered an improvement over parenteral deferoxamine (DFO, Desferal®), particularly in terms of compliance, convenience, patient satisfaction, and health-related quality of life [2, 14–16]. However, barriers still exist to optimal adherence with deferasirox DT, including the need to take the drug in a fasting state, requirements for careful dispersion prior to ingestion, the chalky consistency, and suboptimal gastrointestinal (GI) tolerability [17]. In recognition of the importance of patient adherence to chelation therapy, a film-coated tablet (FCT) formulation for oral administration (either whole or crushed and mixed with soft foods) was developed as an alternative treatment option intended to improve patient acceptability and therefore compliance. Both FCT and DT contain the same active substance (deferasirox) and are administered once daily. Dose is adjusted according to body weight and response to treatment [18–20]. In contrast to the DT, the FCT does not contain the excipients lactose and sodium lauryl sulphate and can be taken with or without a light meal [19, 20] offering a more simple and convenient mode of administration with potential improvements in GI tolerability.

The Phase II, randomized open-label ECLIPSE study demonstrated similar safety profiles for the FCT and DT, including acceptable GI tolerability for both formulations [20]. The study provided evidence that patients receiving the FCT remained on treatment for longer and were more compliant than those receiving the DT. The patient-reported outcome (PRO) analyses also indicated a benefit in favor of the deferasirox FCT, including greater adherence, greater satisfaction, fewer concerns and better palatability [20]. Furthermore, a post-marketing assessment of safety outcomes from data available in the USA (where the FCT became available in April 2015), demonstrated a tendency towards better tolerability with the FCT, particularly in the GI tract [21].

Here, we report analyses from the individual response categories of the various PRO instruments used for the ECLIPSE study, specifically the modified Satisfaction with Iron Chelation Therapy (mSICT) and Palatability Questionnaire items, as well as the GI Symptom Diary. These analyses were performed to better understand the drivers of the improved PROs observed in patients receiving the FCT versus those receiving DT.

## Methods

### Patients

Key inclusion and exclusion criteria have been described elsewhere [20]. In summary, iron overloaded and previously chelated or chelation-naïve male and female patients (aged ≥10 years) with TDT or revised International Prognostic Scoring System very-low, low- or intermediate-risk MDS were enrolled. Key exclusion criteria were: creatinine clearance below contraindication limit as per local label (< 60 mL/min or < 40 mL/min); serum creatinine > 1·5 × upper limit of normal (ULN); alanine aminotransferase > 5 × ULN (unless liver iron concentration confirmed as > 10 mg Fe/g dry weight ≤ 6 months prior to screening); urine protein/urine creatinine ratio > 0·5 mg/mg; or impaired GI function.

### Study design

ECLIPSE was a 24-week, open-label, randomized, multicenter, two-arm, Phase II study that evaluated the deferasirox FCT and DT formulations as previously described [20]. Randomization was stratified by underlying disease and prior chelation treatment. As per protocol, deferasirox DT (taken on an empty stomach, at least 30 min before the next meal) was to be initiated at 20 mg/kg/day in chelation-naïve patients; pre-treated patients were to be initiated at a dose equivalent to their prior chelation treatment (taking conversion rules into account, e.g., 20 mg/kg/day deferasirox equivalent to ~ 40 mg/kg/day DFO) following a 5-day washout period. As per protocol, deferasirox FCT (taken with or after a light meal) was to be initiated at 14 mg/kg/day in chelation-naïve patients; pre-treated patients were to be initiated at a dose equivalent to their prior chelation treatment (taking conversion rules into account, e.g., 20 mg/kg/day DT equivalent to 14 mg/kg/day FCT; conversion factor 1·43) following a 5-day washout period. Dose adjustments to improve treatment response based on serum ferritin levels and the investigator’s judgement were recommended every 4 weeks for chelation-naïve patients, and every 3 months for pre-treated patients, in increments of 5–10 mg/kg/day for DT or 3·5–7 mg/kg/day for FCT, up to a maximum dose of 40 mg/kg/day for DT and 28 mg/kg/day for FCT.

The study was conducted in accordance with Good Clinical Practice guidelines and the Declaration of Helsinki and was approved by independent ethics committees at participating sites. Patients (or parents/guardians) provided written, informed consent prior to enrollment.

### PRO instruments

Evaluation of both deferasirox formulations on patient satisfaction, palatability, and GI symptoms was conducted as a secondary endpoint of the study using PRO questionnaires. Three PRO questionnaires were developed: 1) mSICT; 2) Palatability Questionnaire; and 3) GI Symptoms Diary. Patients completed all PRO questions via a hand-held electronic device either at home (daily diaries) or during scheduled site visits. The instruments underwent full qualitative evaluation [22]; linguistic and psychometric evaluation of the PRO questionnaires was conducted during the trial, supporting the reliability and construct-related validity of the scores [23].

The mSICT questionnaire used five-point response scales to assess adherence (six questions), satisfaction/preference (two questions), and concern domains (three questions). Domain scores were calculated by summing the scores from all questions within the domain; higher scores in adherence (range 6–30) and satisfaction/preference (range 2–10) domains indicated worse outcomes, higher scores in concern domain (range 3–15) indicated fewer concerns. The Palatability Questionnaire consisted of four items: taste and aftertaste of the medication (5-point response scale: 1 = very good to 5 = very bad), whether the medication was taken (i.e., whether the patient vomited after swallowing medication or not) and how the patient perceived the amount of medication to be taken (not enough, just enough, or too much). Overall palatability score ranged from 0 (worst) to 11 (best). These two questionnaires were completed at the start of treatment [SOT], which was week 2 after the first dose (or week 3 if missing), week 3, 13, and end of treatment (within 7 days of the last dose).

The GI Symptom Diary, completed daily, consisted of six items; five items (pain in your belly, nausea, vomiting, constipation, diarrhea) rated on an 11-point scale (0 = best, 10 = worst) and the sixth item, bowel movement frequency during the past 24 h, using seven response options 0 = 0 (none), 1 = 1, 2 = 2, 3 = 3, 4 = 4, 5 = 5–10 and 6 = ≥ 11. The overall GI summary score was calculated from the responses to the five items that used the 11-point scale, ranging from 0 to 50, with higher scores indicating worse symptoms. Item six was analysed as a standalone item.

Summary scores for each of the three PRO questionnaires were also summarized by underlying anemia (thalassemia or MDS), and age categories (10 to < 13, 13 to < 18, 18 to < 50, 50 to < 65 and ≥ 65 years).

### Statistical evaluations

Standard descriptive analyses were performed. No hypothesis was tested. The minimally important difference (MID) was calculated (utilizing ½ standard deviation [SD] [24] and standard error of measurement [25]) for overall scores for the mSICT and palatability summary score, representing the smallest difference between the two treatment groups that is considered clinically important or that implies treatment benefit [26]. MID scores > 1 point for the mSICT domains and palatability scores and MID scores of 0·24 to 0·39 for the GI symptom score indicate a meaningful difference between groups. The full analysis set (FAS) includes all patients randomized to receive treatment, N. The term ‘evaluable patients’ refers to those patients who provided a response to the PRO instruments, as indicated by the investigator. A supportive analysis was conducted to assess the robustness of the PRO results. Missing PRO scores (from mSICT, palatability and GI PRO questionnaire) were imputed using a multiple imputation method under missing at random assumption (MAR, i.e. the probability of missing data is unrelated to the values of PRO score). Given that the missing data patterns observed in the three PRO measures were monotone and intermittent (Table 1), an assessment based on the reasons for missing PRO measures was done, which supported MAR as an acceptable assumption to assess the robustness of the main analysis of PRO measures collected during the trial.

**Table 1:** Completion of the three PRO instruments at each assessment during the study

Week	mSICT		Palatability		GI Symptom Diary*	
	Deferasirox DT *N* = 86 n (%)	Deferasirox FCT *N* = 87 n (%)	Deferasirox DT *N* = 86 n (%)	Deferasirox FCT *N* = 87 n (%)	Deferasirox DT *N* = 86 n (%)	Deferasirox FCT *N* = 87 n (%)
−2					60 (69.8)	59 (67.8)
−1					62 (72.1)	52 (59.8)
1					70 (81.4)	69 (79.3)
2	70 (81.4)	70 (80.5)	69 (80.2)	70 (80.5)	65 (75.6)	71 (81.6)
3	58 (67.4)	51 (58.6)	57 (66.3)	51 (58.6)	64 (74.4)	66 (75.9)
4					60 (69.8)	64 (73.6)
5					58 (67.4)	64 (73.6)
6					64 (74.4)	57 (65.5)
7					57 (66.3)	53 (60.9)
8					59 (68.6)	51 (58.6)
9					55 (64.0)	49 (56.3)
10					53 (61.6)	49 (56.3)
11					51 (59.3)	44 (50.6)
12					51 (59.3)	45 (51.7)
13	59 (68.6)	64 (73.6)	59 (68.6)	62 (71.3)	49 (57.0)	50 (57.5)
14					51 (59.3)	44 (50.6)
15					49 (57.0)	43 (49.4)
16					48 (55.8)	41 (47.1)
17					44 (51.2)	40 (46.0)
18					44 (51.2)	38 (43.7)
19					40 (46.5)	37 (42.5)
20					40 (46.5)	39 (44.8)
21					39 (45.3)	37 (42.5)
22					38 (44.2)	35 (40.2)
23					36 (41.9)	34 (39.1)
24	63 (73.3)	60 (69.0)	63 (73.3)	60 (69.0)	32 (37.2)	26 (29.9)

SOT for the mSICT and palatability were defined as the first PRO assessment at week 2 (or week 3 if missing); for the GI Symptom Diary, week 1 was taken as SOT; ^*^includes patients with at least four complete daily responses. *DT* Dispersible tablet, *FCT* Film-coated tablet, *GI* Gastrointestinal, *PRO* Patient-reported outcome, *mSICT* modified Satisfaction with iron chelation therapy, *SOT* Start of treatment

## Results

### Patient disposition and PRO instrument response

In total, 173 patients from 16 countries worldwide were randomized 1:1 to receive deferasirox DT (*N* = 86) or FCT (*N* = 87) treatment. Overall, 73 (84·9%) and 77 (88·5%) patients in the DT and FCT arm, respectively, completed the treatment period. The treatment groups were balanced with respect to type of anemia and prior chelation therapy, with 70 TDT patients in each arm and 77 and 79 previously chelated patients in the DT and FCT arm, respectively.

**Fig. 1:**
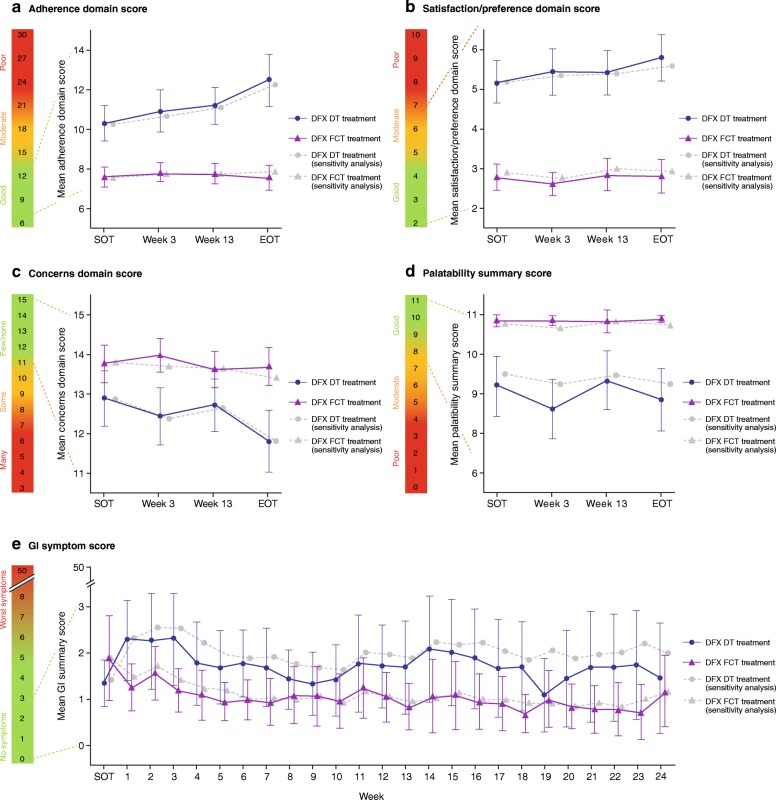
Mean ± 95% CI domain scores for patient-reported outcomes (adherence, satisfaction/preference, and concern) (**a**–**c**), mean palatability score (**d**), and mean gastrointestinal symptom scores (**e**). For adherence (**a**; scale 6–30), satisfaction/preference (**c**; scale 2–10), and GI symptoms (**e**; scale 0–50), higher scores indicate worse outcomes/symptoms. For concern (**c**; scale 3–15) and palatability (**d**; scale 0–11), higher scores indicate fewer concerns and better palatability. **a**–**d**, SOT was defined as week 2 assessment. If missing, then the week 3 assessment was considered SOT. **e**, SOT was defined as week 1 score. If missing, then the week 2 score was considered SOT. CI, confidence interval; DFX, deferasirox; DT, dispersible tablet; EOT, end of treatment; FCT, film-coated tablet; SOT, start of treatment. Figure adapted from Taher AT et al. *Am J Hematology* 2017;92(5):420–428. Published with kind permission of John Wiley & Sons ©2017 The Authors, American Journal of Hematology, Published by Wiley Periodicals, Inc.

A similar proportion of patients completed the three questionnaires for both formulations at the SOT and at the end of treatment (EOT), with the response rate generally lower at the end of the study (Table 1). PRO results obtained using imputed scores, to account for missing PROs, were similar to the main PRO analyses (Fig. 1).

### mSICT and Palatability Questionnaire mean domain results

Completion of these two PRO questionnaires declined from ~ 80% at SOT to 70% at EOT in both treatment arms (Table 1). As previously reported, patients receiving the FCT consistently reported greater adherence, satisfaction/preference and fewer concerns than patients receiving the DT. The difference in scores between treatment groups was > 1 point (MID = 1) at almost all visits for all three domains, indicating a clinically meaningful difference (Fig. 1a, b and c) [20]. Mean palatability summary scores in the FCT treatment arm were consistently high (~ 11) compared with the DT arm (~ 8–9) (Fig. 1d) [20]. The difference in scores between treatment groups was > 1 point (MID = 1), indicating a clinically meaningful difference. In all mean domain scores for the two questionnaires, the 95% confidence intervals did not overlap at most time points analysed. Analysis of summary scores by underlying anemia or age group did not reveal any notable differences compared to the overall findings (see Additional file 1: Table S2).

### Item results from the mSICT adherence domain

To better understand the improved mSICT and Palatability Questionnaire mean domain results with FCT versus DT, patient responses to individual questionnaire items were evaluated. The adherence domain consisted of six questions related to: trouble remembering to take the medication, thinking about stopping the medication, following their doctor’s instructions (including reasons for not always taking the medication, if a patient indicated they did not ‘always’ take their medication as instructed), how hard/easy it was to take the medication, how bothered they were about the time to prepare the medication and the time to wait to eat food. Overall, patients in the FCT arm reported consistent mean adherence scores from SOT (7·6) to EOT (7·5), whereas overall scores in the DT arm worsened by EOT (12·5) compared to SOT (10·3; Fig. 1a) and were slightly higher (indicating worse outcomes).

**Fig. 2:**
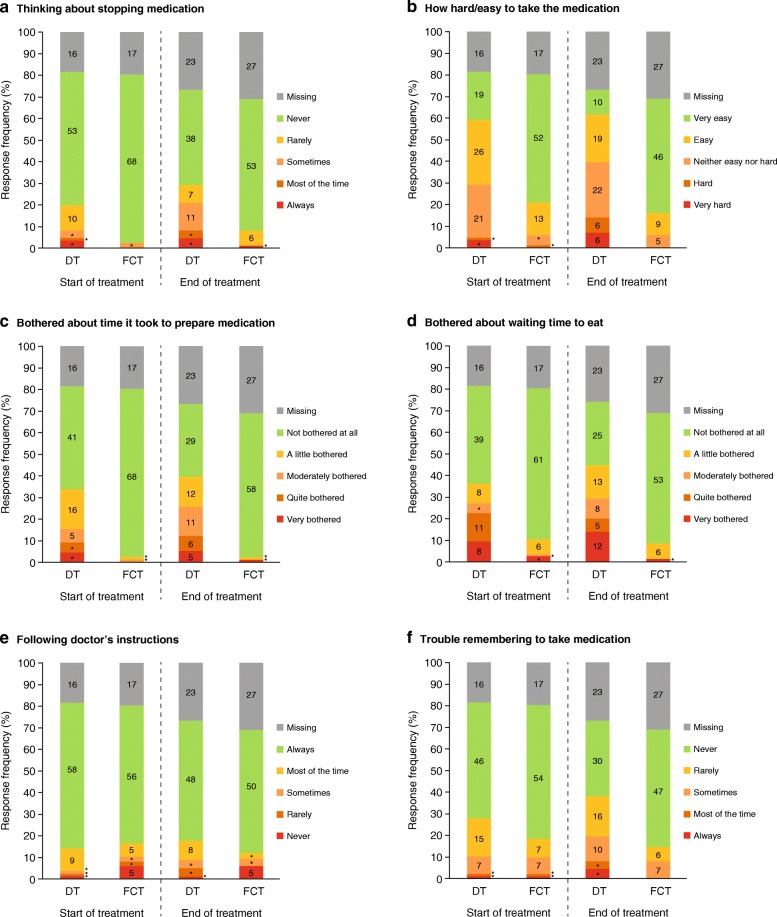
Response frequencies at week 2 (start of treatment) and end of treatment by treatment arm from the mSICT adherence domains, **a** Thinking about stopping medication, **b** How hard/easy to take the medication, **c** Time to prepare medication, **d** Waiting time to eat, **e** Following doctor’s instructions, and **f** Trouble remembering to take medication. Numbers indicate the number of patients in each response category; *n < 5 patients. DT, dispersible tablet; FCT, film-coated tablet; mSICT, modified Satisfaction with Iron Chelation Therapy

Most patients in both treatment arms reported *‘never thinking about stopping medication’* at SOT. By EOT, the proportion of DT recipients who reported *‘never thinking about stopping medication’* was 44·2% (*n* = 38/86 [n = 38/63, 60·3% evaluable patients]) compared with 60·9% (*n* = 53/87 [*n* = 53/60, 88·3% evaluable patients]) of FCT recipients (Fig. 2a). The majority of FCT recipients (*n* = 65/87, 74·7% at SOT [n = 65/70, 92·9% evaluable patients]; *n* = 55/87, 63·2% at EOT [n = 55/60, 91·7% evaluable patients]) found it *‘very easy or easy’* to take their medication, in comparison to DT recipients (*n* = 45/86, 52·3% at SOT [*n* = 45/70, 64·3% evaluable patients]; *n* = 29/86, 33·7% at EOT [n = 29/63, 46·0% evaluable patients]; Fig. 2b). More patients in the FCT group were *‘not bothered at all’* about the time it took to prepare medication (Fig. 2c) or by the time they had to wait to eat (Fig. 2d) than patients in the DT group at both SOT and EOT.

A similar proportion of patients in both treatment arms reported to *‘always’* follow their doctor’s instructions (Fig. 2e), both at SOT (FCT: *n* = 56/87, 64·4% [n = 56/70, 80·0% evaluable patients] and DT: *n* = 58/86, 67·4% [n = 58/70, 82·9% evaluable patients]) and EOT (FCT: *n* = 50/87, 57·5% [*n* = 50/60, 83·3% evaluable patients] and DT: *n* = 48/86, 55·8% [n = 48/63, 76·2% evaluable patients]). In both treatment arms, the proportion of patients *‘rarely or never’* having trouble remembering to take medication was similar (Fig. 2f) at SOT (FCT: *n* = 61/87, 70·1% [n = 61/70, 87·1% evaluable patients] and DT: *n* = 61/86, 70·9% [*n* = 61/70, 87·1% evaluable patients]), declining by EOT in the DT arm (FCT: *n* = 53/87, 60·9% [n = 53/60, 88·3% evaluable patients] and DT: *n* = 46/86, 53·5% [*n* = 46/63, 73·0% evaluable patients]).

### Item results from the mSICT satisfaction domain

The satisfaction domain consisted of three questions related to: satisfaction with how they took the medication, how satisfied/dissatisfied they were with the medication in general, and which type of medication did they like the best (see Overall Preference). Overall, patients in the FCT arm reported consistent mean satisfaction scores from SOT (2·8) to EOT (2·9), whereas overall scores in the DT arm worsened at EOT (5·8) compared to SOT (5·2, Fig. 1b) and were generally higher (indicating worse outcome).

More patients in the FCT than the DT group were ‘*very satisfied’* or ‘*satisfied*’ with taking their medication at SOT (FCT: *n* = 67/87, 77·0% [n = 67/70, 95·7% evaluable patients] and DT: *n* = 32/86, 37·2% [*n* = 32/70, 45·7% evaluable patients]) and EOT (FCT: *n* = 54/87, 62·1% [*n* = 54/60, 90·0% evaluable patients] and DT: *n* = 26/86, 30·2% [*n* = 26/63, 41·3% evaluable patients]). More FCT than DT recipients were also ‘*very satisfied’* or ‘*satisfied*’ with their medication overall at SOT (FCT: *n* = 67/87, 77·0% [*n* = 67/70, 95·7% evaluable patients] and DT: *n* = 40/86, 46·5% [*n* = 40/70, 57·1% evaluable patients]) and EOT (FCT: *n* = 56/87, 64·4% [*n* = 56/60, 93·3% evaluable patients] and DT: *n* = 28/86, 32·6% [*n* = 28/63, 44·4% evaluable patients]).

### Item results from the mSICT concerns domain

**Fig. 3:**
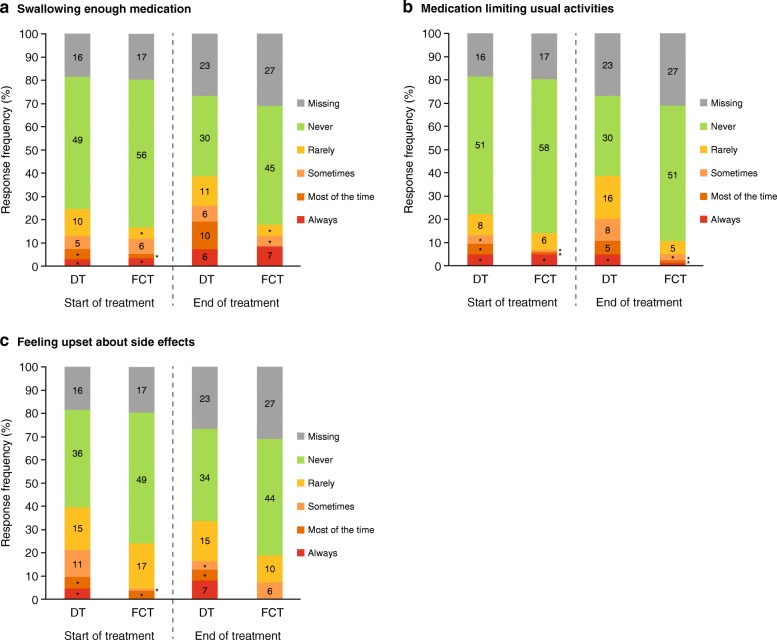
Response frequencies at week 2 (start of treatment) and end of treatment by treatment arm from the mSICT concerns domains, **a** Swallowing enough medication, **b** Medication limiting usual activities, **c** Feeling upset about side effects. Numbers indicate the number of patients in each response category; *n < 5 patients. DT, dispersible tablet; FCT, film-coated tablet; mSICT, modified Satisfaction with Iron Chelation Therapy

The concerns domain consisted of three questions related to: worries about not swallowing enough medication, medication limiting usual activities, and feeling upset about side effects of medication. Overall, patients in the FCT arm reported consistent mean concern scores from SOT (13·8) to EOT (13·7), whereas overall scores in the DT arm worsened at EOT (11·8) compared to SOT (12·9, Fig. 1c) and were generally lower (indicating more concerns). For all questions, there were minimal concerns for both medications at SOT and EOT (Fig. 3a, b and c). There was a general trend for more patients in the FCT than the DT group reporting *‘never’* or *‘rarely’* feeling concerned at SOT and EOT.

### Item results from the Palatability Questionnaire

The Palatability Questionnaire consisted of four questions to assess taste, aftertaste, whether the medication was taken (e.g., swallowed or vomited), and how the patient perceived the amount of medication (liquid) to be taken. Overall, patients in the FCT arm reported consistently high mean palatability scores from SOT (10·8) to EOT (10·9), where a score of 11 was equivalent to the best outcome, whereas overall scores in the DT arm were lower (9·0 at SOT and 8·8 at EOT) and more variable at each time point (Fig. 1d).

**Fig. 4:**
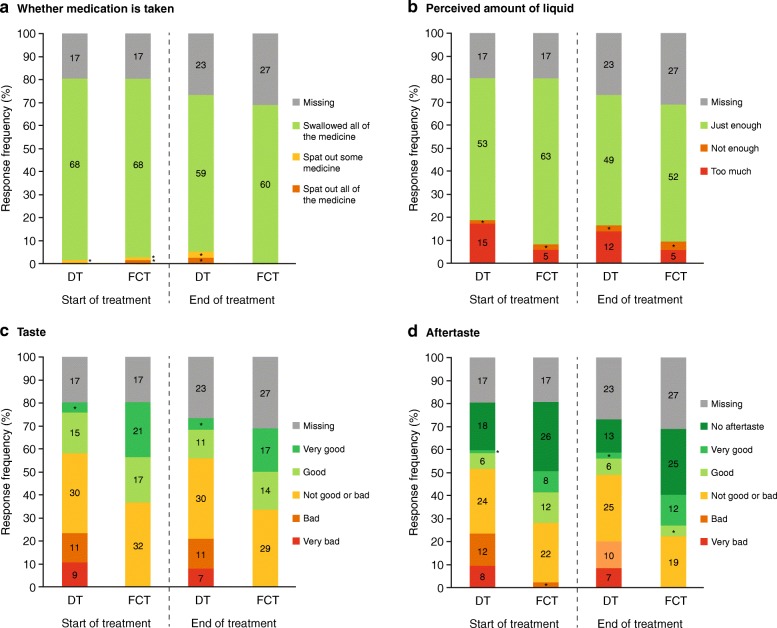
Response frequencies at week 2 (start of treatment) and end of treatment by treatment arm from the Palatability Questionnaire items, **a** Whether medication was taken, **b** Perceived amount of liquid, **c** Taste, and **d** Aftertaste. Numbers indicate the number of patients in each response category; *n < 5 patients; DT, dispersible tablet; FCT, film-coated tablet

Most patients in both the FCT and DT arms swallowed all their medication at SOT (FCT: *n* = 68/87, 78·2% [*n* = 68/70, 97·1% evaluable patients] and DT: *n* = 68/86, 79·1% [*n* = 68/69, 98·6% evaluable patients]) and EOT (FCT: *n* = 60/87, 69·0% [*n* = 60/60, 100·0% evaluable patients] and DT: *n* = 59/86, 68·6% [*n* = 59/63, 93·7% evaluable patients]; Fig. 4a). No patient vomited within 30 min of swallowing their medication. The majority of FCT and DT recipients confirmed that the amount of liquid they took with their medicine for iron overload on that given day was *‘just enough’* both at SOT (FCT: *n* = 63/87, 72·4% [*n* = 63/70, 90·0% evaluable patients] and DT: *n* = 53/86, 61·6% [*n* = 53/69, 76·8% evaluable patients]) and EOT (FCT: *n* = 52/87, 59·8% [*n* = 52/60, 86·7% evaluable patients] and DT: *n* = 49/86, 57·0% [*n* = 49/63, 77·8% evaluable patients]); however, more DT than FCT recipients reported that it was *‘too much’* (SOT: *n* = 15/86, 17·4% [*n* = 15/69, 21·7% evaluable patients] versus *n* = 5/87, 5·8% [*n* = 5/70, 7·1% evaluable patients]; EOT: *n* = 12/86, 14·0% [*n* = 12/63, 19·0% evaluable patients] versus *n* = 5/87, 5·8% [*n* = 5/60, 8·3% evaluable patients]; Fig. 4b). Nearly all patients in the FCT group reported no issue with the taste (100% at SOT and EOT) or aftertaste (98% at SOT and 100% EOT) of their medicine; whereas ~ 20% of patients (~ 30% evaluable patients) at SOT and EOT in the DT group reported that their medication had a *‘bad’* or *‘very bad’* taste or aftertaste (Fig. 4c and d).

### Overall preference

**Fig. 5:**
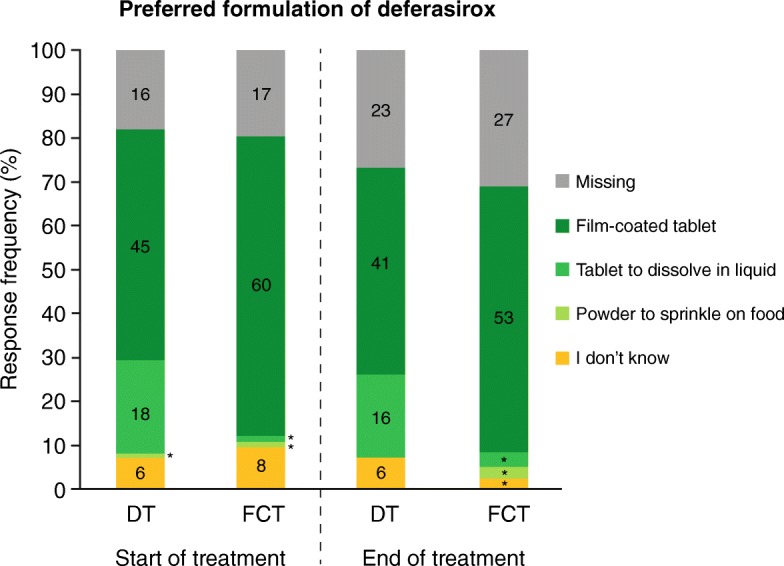
Response frequencies for the preferred formulation of deferasirox at week 2 (start of treatment) and end of treatment by treatment arm. Numbers indicate the number of patients in each response category; *n < 5 patients; DT, dispersible tablet; FCT, film-coated tablet

Evaluation of the preferred formulation of deferasirox was assessed as part of the mSICT satisfaction domain. In patients who received either the DT or the FCT, there was a preference for the FCT (Fig. 5). At EOT, of patients in the FCT arm, 53/87 (60·9% [*n* = 53/60, 88·3% evaluable patients]) reported preference for the FCT, while 3/87 (3·5% [*n* = 3/60, 5·0% evaluable patients]) preferred a DT. Of patients in the DT arm, 41/86 (47·7% [*n* = 41/63, 65·1% evaluable patients]) reported preference for a FCT, while 16/86 (18·6% [*n* = 16/63, 25·4% evaluable patients]) preferred the DT.

### GI Symptom Diary

Completion of the GI Symptom Diary also decreased over the course of the study (Table 1). As previously reported [20], the overall GI symptom scores were low for both formulations over the course of the study (less than 3 out of a maximum of 50; Fig. 1e), indicating that patients experienced very little concern associated with GI symptoms. Analysis of summary scores by underlying anemia or age group also did not reveal any notable differences compared to the overall findings (see Additional file 1: Table S2).

## Discussion

It has been well documented that adherence to iron chelation therapy influences overall survival [2, 4, 27]. Although the introduction of once-daily oral chelation with deferasirox DT was an improvement over parenteral DFO, the properties of deferasirox DT were still not ideal. The deferasirox FCT formulation was specifically developed with the intention of improving patient acceptability, to enhance patient satisfaction and therefore adherence to iron chelation therapy. Besides evaluating the safety profile and pharmacokinetics of the deferasirox FCT relative to the DT formulation, the ECLIPSE trial also examined detailed PRO data to better understand patient preference for, and satisfaction with, the FCT versus the DT. In general, the PROs demonstrated that patients receiving either the FCT or DT had no major issues regarding adherence, preference, satisfaction, palatability, and GI tolerability with their medication, with patients providing mostly neutral or positive responses to the questionnaires. However, patients receiving FCT and DT showed a clear patient preference for the deferasirox FCT over the DT formulation. Furthermore, these findings were maintained irrespective of underlying anemia or age group, indicating consistent outcomes across patient subgroups. As previously reported, the mean domain scores from the mSICT and Palatability Questionnaires showed adherence, preference, satisfaction, and palatability in favor of the deferasirox FCT [20]. Furthermore, the difference in scores between treatment groups was of a magnitude that implies a corresponding treatment benefit (the difference being > 1 point [MID = 1]), indicating a clinically meaningful difference. However, the GI symptom score diaries revealed no clear differences between the two deferasirox formulations, with the majority of patients showing GI disturbances of minimal concern over the 24-week study. This may be attributed to the majority of patients enrolled having experienced prior chelation therapy and having underlying thalassemia, in whom GI disturbances are usually mild-to-moderate [28]. Nevertheless, the analyses presented here show that item results from the mSICT and Palatability Questionnaires provide several specific underlying factors that can be considered as reasons for patients preferring the deferasirox FCT to a degree that may improve their clinical outcomes.

The adherence domain outcomes from the mSICT showed that all FCT and DT patients generally followed their doctor’s instructions when taking their medication, had minimal trouble remembering to take their medication, and rarely thought about stopping. However, by EOT a slightly lower proportion of patients in the DT arm reported *‘never or rarely’* having trouble remembering to take their medication compared to the FCT arm, as well as *‘never’* thinking about stopping. The finding that the majority of patients receiving the FCT formation found it *‘easy’* or *‘very easy’* to take their medication compared with those in the DT arm, along with not being bothered by the time it took to take their medication or waiting to eat, suggests that the FCT may provide a more convenient mode of administration. The FCT can be taken either on an empty stomach or with a low-fat meal (< 7% fat content and ~ 250 cal) [19], whereas it is recommended that the DT is taken only on an empty stomach at least 30 min before the next meal, following dispersion in a glass of water, orange juice or apple juice [18], which takes approximately 3 min to prepare. Previous studies have indicated that the ability to take deferasirox with food, at breakfast in particular, is important to patients [17]. The mode and convenience of administration of the FCT also may have contributed to the majority of patients feeling satisfied with their medication and experiencing fewer concerns, such as worry over swallowing enough medication or impact on their usual activities. Further study is warranted to corroborate these findings.

Patient responses to items on the Palatability Questionnaire revealed similar trends as those observed for the mSICT items, whereby patients receiving the FCT trended towards reporting more positive response. Some patients receiving the DT reported that it tasted *‘bad’* or *‘very bad.’* However minimal issues with taste or aftertaste were reported by patients receiving the FCT. The majority of FCT and DT recipients also confirmed that the amount of liquid they took with their medicine was *‘just enough’.* On the other hand, more DT recipients compared to FCT recipients indicated that the amount of liquid was *‘too much’,* because of the need to disperse their medication in ~ 200 mL (for doses ≥1 g, ~ 100 mL for doses < 1 g). Taking all these factors into account, adherence, satisfaction, concerns, and palatability, there was a clear preference for the FCT over the DT both at week 2 (SOT) and EOT.

This study was limited by the short duration of follow-up – 24 weeks is a relatively short period of time in patients receiving chronic treatment for iron overload that can last a lifetime – however, apparent differences were notable between the two formulations both at 2 weeks (SOT) and at EOT. In addition, not all patients completed the various PRO instruments (attributed to missed patient visits, patients forgetting to bring their PRO devices or oversight in completing the questionnaires during site visits), which may have led to some bias in the findings. However, the completion rate was similar for both formulations and as expected in a PRO study, with a decline in response rate over the course of the study, particularly for the GI questionnaire. This may have been the result of the requirement for daily completion during the study, which some patients may have found burdensome. It should also be noted that the completion rates were based on the FAS and were not adjusted to account for decreasing numbers of patients (discontinuations) over the 24-week study. Furthermore, this was a descriptive, exploratory study. Additional long-term experiences from patients in clinical practice treated with the FCT, as well as other clinical trials with longer treatment duration, are required to confirm whether the improved satisfaction, preference, and adherence shown for FCT are maintained over longer time periods. With a short-term safety profile consistent with the known deferasirox DT [20] – well characterized in an extensive clinical trial program – longer follow-up of patients receiving the FCT will also help determine whether the improvements in PROs will translate into better clinical outcomes. In particular, patients switching from the DT to the FCT will require careful monitoring to avoid potential ‘over-chelation’ with improved adherence, even with the initial FCT dose reduction (e.g., 20 mg/kg/day DT equivalent to 14 mg/kg/day FCT; conversion factor 1·43).

## Conclusions

In conclusion, for all patients regardless of age group or underlying disease, these findings from the detailed analyses of responses to various validated PRO instruments could suggest a preference in favor of the once-daily deferasirox FCT formulation. The FCT, which contains the same active ingredient as the DT but with excipients removed, and can be taken orally once daily with or without a light meal, may therefore be less burdensome to patients than the DT formulation. Together with a safety profile consistent with the known deferasirox DT formulation, as previously reported [20], the deferasirox FCT may improve patient experience with iron chelation therapy resulting in greater adherence. It is anticipated that better adherence will translate into a reduction in the frequency and severity of iron overload-related complications.

## Additional file

Additional file 1: **Table S1.** Summary of domain scores for the mSICT and palatability PRO instruments, by week, treatment and underlying anemia or age group. **Table S2.** Summary of domain scores for the GI Symptom Diary, by week, treatment and underlying anemia or age group. (DOCX 45 kb)

## Abbreviations

CI: Confidence interval
DFO: Deferoxamine
DFX: Deferasirox
DT: Dispersible tablet
EOT: End of treatment
FAS: Full analysis set
FCT: Film-coated tablet
GI: Gastrointestinal
MDS: Myelodysplastic syndromes
MID: Minimally important difference
mSICT: Modified satisfaction with iron chelation therapy
PRO: Patient-reported outcome
SOT: Start of treatment
TDT: Transfusion-dependent thalassemia
ULN: Upper limit of normal
