# Supplementary material for: Patient-reported outcomes from a randomized phase II study of the deferasirox film-coated tablet in patients with transfusion-dependent anemias
Source: Health Qual Life Outcomes. 2018 Nov 19;16:216. doi: 10.1186/s12955-018-1041-5 (PMC6245526; doi:10.1186/s12955-018-1041-5)
Supplement: Supplementary file 1 — Table S1. Summary of domain scores for the mSICT and palatability PRO instruments, by week, treatment and underlying anemia or age group. Table S2. Summary of domain scores for the GI Symptom Diary, by week, treatment and underlying anemia or age group. (DOCX 45 kb) [file 12955_2018_1041_MOESM1_ESM.docx]

# Additional file

**Table S1** Summary of domain scores for the mSICT and palatability PRO instruments, by week, treatment and underlying anemia or age group. For adherence (scale 6–30) and satisfaction/preference (scale 2–10) higher scores indicate worse outcomes/symptoms. For concern (scale 3–15) and palatability (scale 0–11), higher scores indicate fewer concerns and better palatability. Start of treatment for the mSICT and palatability were defined as the first PRO assessment at week 2 (or week 3 if missing). DT, dispersible tablet; FCT, film-coated tablet; mSCIT, modified Satisfaction with Iron Chelation Therapy; PRO, patient-reported outcome

Type of anemia: Myelodysplastic syndromes

|  |  |  | **mSICT domains** | | | **Palatability** |  |
| --- | --- | --- | --- | --- | --- | --- | --- |
| **Visit** | **Treatment** | **Statistics** | **Adherence** | **Satisfaction/ preference** | **Concern** |  | |
| Start of treatment | DT (N = 16) | n | 11 | 11 | 11 | 10 | |
|  |  | Median [Min, Max] | 7 [6, 11] | 4 [2, 7] | 13 [3, 15] | 11 [4, 11] | |
|  | FCT (N = 16) | n | 9 | 9 | 9 | 9 | |
|  |  | Median [Min, Max] | 7 [6, 9] | 4 [2, 6] | 15 [13, 15] | 11 [11, 11] | |
| Week 3 | DT (N = 16) | n | 8 | 8 | 8 | 8 | |
|  |  | Median [Min, Max] | 7 [6, 9] | 4 [2, 6] | 14·5 [6, 15] | 11 [11, 11] | |
|  | FCT (N = 16) | n | 6 | 6 | 6 | 6 | |
|  |  | Median [Min, Max] | 7 [6, 11] | 4 [2, 6] | 15 [12, 15] | 11 [11, 11] | |
| Week 13 | DT (N = 16) | n | 8 | 8 | 8 | 8 | |
|  |  | Median [Min, Max] | 8·5 [7, 15] | 4 [2, 8] | 14 [6, 15] | 11 [11, 11] | |
|  | FCT (N = 16) | n | 9 | 9 | 9 | 9 | |
|  |  | Median [Min, Max] | 7 [6, 8] | 4 [2, 7] | 15 [13, 15] | 11 [11, 11] | |
| End of treatment | DT (N = 16) | n | 9 | 9 | 9 | 9 | |
|  |  | Median [Min, Max] | 7 [6, 10] | 4 [4, 8] | 15 [10, 15] | 11 [4, 11] | |
|  | FCT (N = 16) | n | 11 | 11 | 11 | 11 | |
|  |  | Median [Min, Max] | 6 [6, 12] | 4 [2, 7] | 14 [10, 15] | 11 [10, 11] | |

Type of anemia: Thalassemia

|  |  |  | **mSICT domains** | | | **Palatability** |  |
| --- | --- | --- | --- | --- | --- | --- | --- |
| **Visit** | **Treatment** | **Statistics** | **Adherence** | **Satisfaction/ preference** | **Concern** |  | |
| Start of treatment | DT (N = 70) | n | 59 | 59 | 59 | 59 | |
|  |  | Median [Min, Max] | 10 [6, 22] | 6 [2, 10] | 14 [3, 15] | 11 [2, 11] | |
|  | FCT (N = 70) | n | 60 | 60 | 60 | 60 | |
|  |  | Median [Min, Max] | 7 [6, 14] | 2 [2, 10] | 15 [7, 15] | 11 [8, 11] | |
| Week 3 | DT (N = 70) | n | 50 | 50 | 50 | 49 | |
|  |  | Median [Min, Max] | 10·5 [6, 21] | 6 [2, 10] | 13 [5, 15] | 10 [4, 11] | |
|  | FCT (N = 70) | n | 44 | 44 | 44 | 44 | |
|  |  | Median [Min, Max] | 7 [6, 14] | 2 [2, 6] | 15 [9, 15] | 11 [9, 11] | |
| Week 13 | DT (N = 70) | n | 51 | 51 | 51 | 51 | |
|  |  | Median [Min, Max] | 11 [6, 23] | 6 [2, 10] | 13 [7, 15] | 11 [4, 11] | |
|  | FCT (N = 70) | n | 54 | 54 | 54 | 52 | |
|  |  | Median [Min, Max] | 7 [6, 13] | 2 [2, 10] | 14 [6, 15] | 11 [2, 11] | |
| End of treatment | DT (N = 70) | n | 54 | 54 | 54 | 54 | |
|  |  | Median [Min, Max] | 13 [6, 28] | 6 [2, 10] | 12 [3, 15] | 11 [2, 11] | |
|  | FCT (N = 70) | n | 48 | 48 | 48 | 48 | |
|  |  | Median [Min, Max] | 6 [6, 19] | 2 [2, 10] | 15 [8, 15] | 11 [10, 11] | |

Age category (years): 10 to <13

|  |  |  | **mSICT domains** | | | **Palatability** |
| --- | --- | --- | --- | --- | --- | --- |
| **Visit** | **Treatment** | **Statistics** | **Adherence** | **Satisfaction/ preference** | **Concern** |  |
| Start of treatment | DT (N = 2) | n | 2 | 2 | 2 | 2 |
|  |  | Median [Min, Max] | 10·5 [6, 15] | 2 [2, 2] | 15 [15, 15] | 8 [5, 11] |
|  | FCT (N = 2) | n | 2 | 2 | 2 | 2 |
|  |  | Median [Min, Max] | 7·5 [6, 9] | 3 [2, 4] | 14 [13, 15] | 10·5 [10, 11] |
| Week 3 | DT (N = 2) | n | 2 | 2 | 2 | 2 |
|  |  | Median [Min, Max] | 9·5 [6, 13] | 2·5 [2, 3] | 14·5 [14, 15] | 10·5 [10, 11] |
|  | FCT (N = 2) | n | 2 | 2 | 2 | 2 |
|  |  | Median [Min, Max] | 10·5 [10, 11] | 4 [3, 5] | 13·5 [12, 15] | 10 [10, 10] |
| Week 13 | DT (N = 2) | n | 1 | 1 | 1 | 1 |
|  |  | Median [Min, Max] | 11 | 2 | 14 | 11 |
|  | FCT (N = 2) | n | 2 | 2 | 2 | 2 |
|  |  | Median [Min, Max] | 8 [7, 9] | 2·5 [2, 3] | 14·5 [14, 15] | 11 [11, 11] |
| End of treatment | DT (N = 2) | n | 1 | 1 | 1 | 1 |
|  |  | Median [Min, Max] | 10 | 2 | 15 | 11 |
|  | FCT (N = 2) | n | 1 | 1 | 1 | 1 |
|  |  | Median [Min, Max] | 6 | 2 | 15 | 10 |

Age category (years): 13 to <18

|  |  |  | **mSICT domains** | | | **Palatability** |  |
| --- | --- | --- | --- | --- | --- | --- | --- |
| **Visit** | **Treatment** | **Statistics** | **Adherence** | **Satisfaction/ preference** | **Concern** |  | |
| Start of treatment | DT (N = 8) | n | 8 | 8 | 8 | 8 | |
|  |  | Median [Min, Max] | 10 [7, 14] | 4 [2, 7] | 14 [9, 15] | 11 [5, 11] | |
|  | FCT (N = 9) | n | 7 | 7 | 7 | 7 | |
|  |  | Median [Min, Max] | 7 [6, 13] | 2 [2, 4] | 15 [7, 15] | 11 [8, 11] | |
| Week 3 | DT (N = 8) | n | 7 | 7 | 7 | 7 | |
|  |  | Median [Min, Max] | 9 [8, 14] | 4 [2, 6] | 13 [11, 15] | 11 [4, 11] | |
|  | FCT (N = 9) | n | 5 | 5 | 5 | 5 | |
|  |  | Median [Min, Max] | 9 [6, 14] | 2 [2, 6] | 14 [9, 15] | 11 [10, 11] | |
| Week 13 | DT (N = 8) | n | 8 | 8 | 8 | 8 | |
|  |  | Median [Min, Max] | 11·5 [8, 13] | 5 [2, 6] | 12·5 [8, 15] | 11 [5, 11] | |
|  | FCT (N = 9) | n | 8 | 8 | 8 | 7 | |
|  |  | Median [Min, Max] | 9 [6, 12] | 2 [2, 4] | 14·5 [8, 15] | 11 [2, 11] | |
| End of treatment | DT (N = 8) | n | 8 | 8 | 8 | 8 | |
|  |  | Median [Min, Max] | 13·5 [8, 18] | 5 [3, 10] | 13 [7, 15] | 11 [5, 11] | |
|  | FCT (N = 9) | n | 4 | 4 | 4 | 4 | |
|  |  | Median [Min, Max] | 9·5 [8, 19] | 2 [2, 2] | 15 [12, 15] | 10·5 [10, 11] | |

Age category (years): 18 to <50

|  |  |  | **mSICT domains** | | | **Palatability** |  |
| --- | --- | --- | --- | --- | --- | --- | --- |
| **Visit** | **Treatment** | **Statistics** | **Adherence** | **Satisfaction/ preference** | **Concern** |  | |
| Start of treatment | DT (N = 59) | n | 48 | 48 | 48 | 48 | |
|  |  | Median [Min, Max] | 9·5 [6, 22] | 6 [2, 10] | 14 [3, 15] | 11 [2, 11] | |
|  | FCT (N = 59) | n | 51 | 51 | 51 | 51 | |
|  |  | Median [Min, Max] | 7 [6, 14] | 2 [2, 10] | 15 [7, 15] | 11 [9, 11] | |
| Week 3 | DT (N = 59) | n | 40 | 40 | 40 | 39 | |
|  |  | Median [Min, Max] | 11 [6, 21] | 6 [2, 10] | 12·5 [5, 15] | 10 [4, 11] | |
|  | FCT (N = 59) | n | 37 | 37 | 37 | 37 | |
|  |  | Median [Min, Max] | 7 [6, 12] | 2 [2, 4] | 15 [9, 15] | 11 [9, 11] | |
| Week 13 | DT (N = 59) | n | 41 | 41 | 41 | 41 | |
|  |  | Median [Min, Max] | 11 [6, 23] | 6 [2, 10] | 14 [7, 15] | 11 [4, 11] | |
|  | FCT (N = 59) | n | 44 | 44 | 44 | 43 | |
|  |  | Median [Min, Max] | 7 [6, 13] | 2 [2, 10] | 14 [6, 15] | 11 [10, 11] | |
| End of treatment | DT (N = 59) | n | 44 | 44 | 44 | 44 | |
|  |  | Median [Min, Max] | 13 [6, 28] | 6 [2, 10] | 12 [3, 15] | 10 [2, 11] | |
|  | FCT (N = 59) | n | 43 | 43 | 43 | 43 | |
|  |  | Median [Min, Max] | 6 [6, 13] | 2 [2, 10] | 15 [8, 15] | 11 [10, 11] | |

Age category (years): 50 to <65

|  |  |  | **mSICT domains** | | | **Palatability** |  |
| --- | --- | --- | --- | --- | --- | --- | --- |
| **Visit** | **Treatment** | **Statistics** | **Adherence** | **Satisfaction/ preference** | **Concern** |  | |
| Start of treatment | DT (N = 5) | n | 2 | 2 | 2 | 2 | |
|  |  | Median [Min, Max] | 9·5 [7, 12] | 4 [4, 4] | 13·5 [12, 15] | 11 [11, 11] | |
|  | FCT (N = 3) | n | 1 | 1 | 1 | 1 | |
|  |  | Median [Min, Max] | 6 | 3 | 15 | 11 | |
| Week 3 | DT (N = 5) | n | 2 | 2 | 2 | 2 | |
|  |  | Median [Min, Max] | 9 [9, 9] | 5 [4, 6] | 12 [11, 13] | 11 [11, 11] | |
|  | FCT (N = 3) | n | 1 | 1 | 1 | 1 | |
|  |  | Median [Min, Max] | 6 | 2 | 15 | 11 | |
| Week 13 | DT (N = 5) | n | 2 | 2 | 2 | 2 | |
|  |  | Median [Min, Max] | 9·5 [9, 10] | 5 [4, 6] | 12·5 [12, 13] | 11 [11, 11] | |
|  | FCT (N = 3) | n | 1 | 1 | 1 | 1 | |
|  |  | Median [Min, Max] | 8 | 4 | 15 | 11 | |
| End of treatment | DT (N = 5) | n | 2 | 2 | 2 | 2 | |
|  |  | Median [Min, Max] | 10 [10, 10] | 5 [4, 6] | 13 [11, 15] | 11 [11, 11] | |
|  | FCT (N = 3) | n | 1 | 1 | 1 | 1 | |
|  |  | Median [Min, Max] | 8 | 4 | 15 | 11 | |

Age category (years): ≥65

|  |  |  | **mSICT domains** | | | **Palatability** |  |
| --- | --- | --- | --- | --- | --- | --- | --- |
| **Visit** | **Treatment** | **Statistics** | **Adherence** | **Satisfaction/ preference** | **Concern** |  | |
| Start of treatment | DT (N = 12) | n | 10 | 10 | 10 | 9 | |
|  |  | Median [Min, Max] | 6·5 [6, 11] | 4 [2, 7] | 12·5 [3, 15] | 11 [4, 11] | |
|  | FCT (N = 14) | n | 9 | 9 | 9 | 9 | |
|  |  | Median [Min, Max] | 7 [6, 9] | 4 [2, 6] | 15 [13, 15] | 11 [11, 11] | |
| Week 3 | DT (N = 12) | n | 7 | 7 | 7 | 7 | |
|  |  | Median [Min, Max] | 7 [6, 9] | 4 [2, 4] | 15 [6, 15] | 11 [11, 11] | |
|  | FCT (N = 14) | n | 6 | 6 | 6 | 6 | |
|  |  | Median [Min, Max] | 7 [6, 11] | 4 [3, 6] | 14·5 [12, 15] | 11 [11, 11] | |
| Week 13 | DT (N = 12) | n | 7 | 7 | 7 | 7 | |
|  |  | Median [Min, Max] | 8 [7, 15] | 4 [2, 8] | 14 [6, 15] | 11 [11, 11] | |
|  | FCT (N = 14) | n | 9 | 9 | 9 | 9 | |
|  |  | Median [Min, Max] | 7 [6, 8] | 5 [2, 7] | 15 [13, 15] | 11 [11, 11] | |
| End of treatment | DT (N = 12) | n | 8 | 8 | 8 | 8 | |
|  |  | Median [Min, Max] | 7 [6, 9] | 4 [4, 8] | 15 [10, 15] | 11 [4, 11] | |
|  | FCT (N = 14) | n | 11 | 11 | 11 | 11 | |
|  |  | Median [Min, Max] | 6 [6, 12] | 4 [2, 7] | 14 [10, 15] | 11 [10, 11] | |

**Table S2** Summary of domain scores for the GI symptom diary, by week, treatment and underlying anemia or age group. For GI symptoms (scale 0–50), higher scores indicate worse outcomes/symptoms. Start of treatment was defined as week 1 score; if missing, then the week 2 score was considered. DT, dispersible tablet; FCT, film-coated tablet, GI, gastrointestinal

| **Visit** | **Treatment** | **Statistics** | **MDS** | **Thalassemia** | **10 to < 13 years** | **13 to < 18 years** | **18 to < 50 years** | **50 to < 65 years** | **≥65 years** |
| --- | --- | --- | --- | --- | --- | --- | --- | --- | --- |
| Start of treatment | DT | n | 9 | 60 | 2 | 7 | 50 | 3 | 7 |
|  |  | Median  [Min, Max] | 1·0  [0, 5·5] | 0  [0, 8·3] | 1·1  [0, 2·2] | 0·0  [0, 3·3] | 0·0  [0, 8·3] | 4·7  [2·0, 5·4] | 0·0  [0, 5·5] |
|  | FCT | n | 12 | 52 | 2 | 6 | 44 | 3 | 10 |
|  |  | Median  [Min, Max] | 0·1  [0, 7·3] | 0·3  [0, 22·2] | 0·1  [0, 0·2] | 0·2  [0, 7·8] | 0·5 [0, 22·2] | 0·0  [0, 7·3] | 0·1  [0, 4·8] |
| Week 4 | DT | n | 14 | 46 | 1 | 6 | 38 | 5 | 10 |
|  |  | Median  [Min, Max] | 0·4  [0, 9·7] | 0·4  [0, 20·0] | 0·0 | 1·0  [0, 8·3] | 0·2  [0, 20·0] | 0·.8 [0·3, 6·2] | 0·1  [0, 9·7] |
|  | FCT | n | 13 | 50 | 2 | 6 | 42 | 3 | 11 |
|  |  | Median  [Min, Max] | 0·8  [0, 9·4] | 0·2  [0, 10·8] | 0·1  [0, 0·1] | 0·1  [0, 2·8] | 0·3  [0, 10·8] | 0·8  [0, 3·9] | 0·3  [0, 9·4] |
| Week 8 | DT | n | 12 | 47 | 1 | 7 | 38 | 5 | 8 |
|  |  | Median  [Min, Max] | 0·4  [0, 3·5] | 0·0  [0, 8·4] | 0·0 | 0·0  [0, 1·5] | 0·3  [0, 8·4] | 1·0  [0, 7·2] | 0·4  [0, 3·5] |
|  | FCT | n | 14 | 36 | 1 | 1 | 34 | 3 | 12 |
|  |  | Median  [Min, Max] | 1·0 [0, 10·6] | 0·0  [0, 4·3] | 0·0 | 0·3 | 0·0  [0, 4·3] | 1·8  [0·1, 4·9] | 0·4  [0, 10·6] |
| Week 12 | DT | n | 10 | 41 | – | 7 | 33 | 3 | 8 |
|  |  | Median  [Min, Max] | 1·3  [0, 3·7] | 0·0  [0, 13·2] | – | 0·0  [0, 0·7] | 0·0  [0, 13·2] | 2·7  [1·8, 9·6] | 0·6  [0, 3·7] |
|  | FCT | n | 14 | 30 | 1 | 1 | 28 | 3 | 12 |
|  |  | Median  [Min, Max] | 1·2  [0, 6·7] | 0·0  [0, 3·7] | 1·1 | 0·0 | 0·0  [0, 3·7] | 5·4  [0·9, 6·7] | 0·6  [0, 5·7] |
| Week 16 | DT | n | 10 | 38 | 1 | 7 | 29 | 3 | 8 |
|  |  | Median  [Min, Max] | 0·2 [0, 1·8] | 0·3  [0, 19·4] | 0·0 | 0·0  [0, 0·4] | 0·8  [0, 19·4] | 1·8  [1·6, 11·8] | 0·0  [0, 0·6] |
|  | FCT | n | 11 | 29 | 1 | 3 | 25 | 2 | 10 |
|  |  | Median  [Min, Max] | 0·6  [0, 8·8] | 0·0  [0, 4·0] | 0·0 | 0·0  [0, 0] | 0·0  [0, 4·0] | 2·9 [0·6, 5·2] | 0·3  [0, 8·8] |
| Week 20 | DT | n | 9 | 31 | 1 | 6 | 23 | 3 | 7 |
|  |  | Median  [Min, Max] | 0·0  [0, 4·1] | 0·0  [0, 16·0] | 0·0 | 0·2  [0, 2·0] | 0·0 [0, 16·0] | 4·1  [0·1, 9·7] | 0·0  [0, 0·0] |
|  | FCT | n | 12 | 26 | - | 1 | 25 | 2 | 11 |
|  |  | Median  [Min, Max] | 0·9  [0, 5·4] | 0·0  [0, 1·7] | - | 0·2 | 0·0  [0, 1·7] | 2·6  [0·2, 5·0] | 0·7  [0, 5·4] |
| Week 24 | DT | n | 9 | 23 | - | 5 | 17 | 3 | 7 |
|  |  | Median  [Min, Max] | 0·1  [0, 3·6] | 0·0  [0, 13·0] | - | 0·0  [0, 1·3] | 0·0  [0, 13·0] | 3·6  [2·7, 9·5] | 0·0  [0, 1·0] |
|  | FCT | n | 10 | 15 | - | 1 | 14 | 1 | 10 |
|  |  | Median  [Min, Max] | 0·5  [0, 6·9] | 0·1  [0, 4·0] | – | 0·1 | 0·1  [0, 4·0] | 0·0 | 1·7  [0, 6·9] |
